# Mycobacteria develop biofilms on airway epithelial cells and promote mucosal barrier disruption

**DOI:** 10.1016/j.isci.2024.111063

**Published:** 2024-09-27

**Authors:** Amy M. Barclay, Dennis K. Ninaber, Ronald W.A. L. Limpens, Kimberley V. Walburg, Montserrat Bárcena, Pieter S. Hiemstra, Tom H.M. Ottenhoff, Anne M. van der Does, Simone A. Joosten

**Affiliations:** 1 Leiden University Center for Infectious Diseases, (LUCID), Leiden University Medical Center, Leiden, the Netherlands; 2 PulmoScience Lab, Department of Pulmonology, Leiden University Medical Center, Leiden, the Netherlands; 3 Department of Cell and Chemical Biology, Leiden University Medical Center, Leiden, the Netherlands

**Keywords:** Microbiology, Bacteriology, Medical microbiology, Clinical microbiology

## Abstract

Tuberculosis displays several features commonly linked to biofilm-associated infections, including recurrence of infection and resistance to antibiotic treatment. The respiratory epithelium represents the first line of defense against pathogens such as *Mycobacterium tuberculosis* (Mtb). Here, we use an air-liquid interface model of human primary bronchial epithelial cells (PBEC) to explore the capability of four species of mycobacteria (Mtb, *M. bovis* (BCG), *M. avium,* and *M. smegmatis*) to form biofilms on airway epithelial cells. Mtb, BCG, and *M. smegmatis* consistently formed biofilms with extracellular matrixes on PBEC cultures. Biofilms varied in biomass, matrix polysaccharide content, and bacterial metabolic activity between species. Exposure of PBEC to mycobacteria caused the disruption of the epithelial barrier and was accompanied by mostly apical non-apoptotic cell death. Structural analysis revealed pore-like structures in 7-day biofilms. Taken together, mycobacteria can form biofilms on human airway epithelial cells, and long-term infection negatively affects barrier function and promotes cell death.

## Highlights

• Mycobacteria, including Mtb, can form biofilms on bronchial epithelial cells
• Mycobacteria can disrupt epithelial barrier integrity and promote cell death
• Mycobacterial biofilm matrixes are polysaccharide-rich
• 7-day biofilms develop pore-like structures

## Graphical abstract

**Figure:**
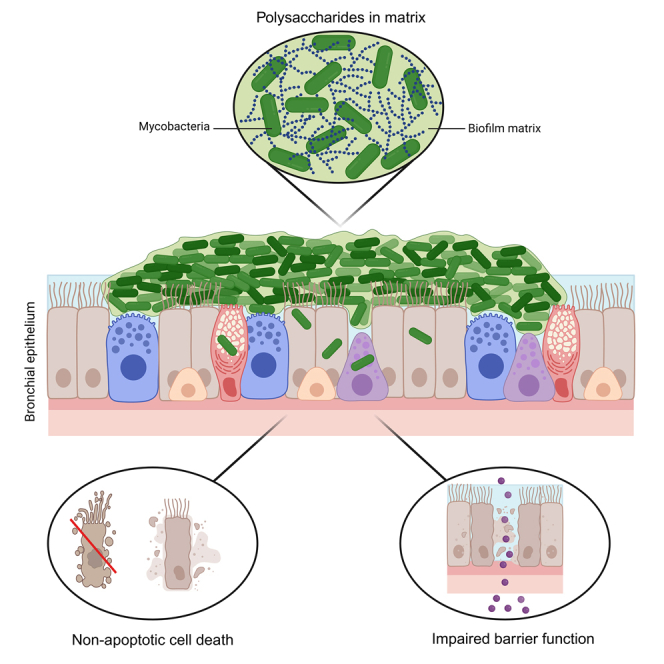

## Introduction

*Mycobacterium tuberculosis* (Mtb), the causative agent of tuberculosis (TB), is a highly successful respiratory pathogen which has infected a quarter of the world population and claims over 1.5 million lives per year.^1^ Although Mtb is typically described as an intracellular pathogen, TB displays several features that are commonly linked to biofilm-associated infections. These include recurrence of infection, and resistance to antibiotic treatment and host immunity.

Biofilms are communities of bacteria enclosed in a self-produced extracellular matrix that adhere to biotic or abiotic surfaces, or form aggregates not attached to a surface.^2^ The biofilm matrix contains substances such as polysaccharides, lipids, proteins, and extracellular DNA, that together encapsulate the bacteria. Biofilm formation shields bacteria from environmental stressors such as antibiotics and facilitates horizontal gene transfer. For example, it has been shown that plasmid conjugation is up to 700 times more efficient in biofilms of *Escherichia coli* compared to planktonic bacteria.^3^ In addition, biofilms are able to subvert host immune responses via several mechanisms.^4^ For example, the high biomass of staphylococcal biofilms dilutes targeting antibodies and thereby interferes with opsonization.^5^ *Staphylococcus aureus* biofilms can also attenuate host inflammatory responses by skewing macrophage polarization in mice.^6^ Furthermore, bacteria residing in biofilms often exhibit an altered phenotype compared to planktonic bacteria. For example, they may have drastically reduced growth rates, leading to the formation of dormant or persister cells,^7^ which is a major cause of reduced susceptibility to antibiotics targeting the DNA replication machinery. Taken together, biofilms are considered an extremely effective bacterial survival strategy.

There is limited knowledge about biofilm formation by mycobacteria, particularly *in vivo* and on biotic surfaces, though there are some indications that suggest the existence of such biofilms. The rope-like aggregation of many species of mycobacteria *in vivo* and *in vitro*, referred to as cording,^8^ is a known virulence factor. It may also point to a tendency to form biofilms, as bacteria aggregate in an organized manner.^9^^,^^10^ Another hallmark of TB is the formation of granulomas, which upon cavitation can release substantial numbers of Mtb bacilli, also into the lumen of the lungs. This, together with the findings that necrotizing lesions are hypoxic and drug tolerant persisters are present, supports the hypothesis that Mtb may grow as a biofilm in such (cavitating) lesions.^11^ In a previous study using lung sections of patients who succumbed to TB, Mtb bacilli were found to co-localize with a cellulose-rich matrix, which is suggestive of biofilm formation.^12^ Moreover, other mycobacterial species including *M. bovis* (BCG), *M. avium*, *M. smegmatis*, and *M. marinum* were capable of biofilm formation *in vitro,* mostly on abiotic surfaces.^13^^,^^14^^,^^15^^,^^16^^,^^17^^,^^18^^,^^19^ However, a direct analysis of the ability of various mycobacteria to form biofilms on relevant human biotic surfaces, as well as a comparative analysis between various mycobacterial species is lacking.

Epithelial cells line the inside of the respiratory tract and therefore represent a major part of the microenvironment for Mtb and other mycobacteria. They are likely among the first cells to encounter inhaled mycobacteria during first exposure and may re-encounter mycobacteria upon rupture of a granuloma. Both alveolar and airway epithelial cells have been shown to become infected by mycobacteria.^20^ In addition, we have previously shown that bronchial epithelial cells rapidly mount a general host response to mycobacterial infection which consists of upregulation of antimicrobials, and increased secretion of inflammatory cytokines and chemokines which directly attract neutrophils.^21^ However, the effects of biofilm formation on the mucosal barrier have not been thoroughly investigated. For example, lung epithelial cell viability and barrier integrity upon infection remain unknown. In addition, the capacity of host defense mechanisms such as mucociliary clearance to prevent mycobacterial biofilm formation has not yet been elucidated.

Herein, we utilized well-differentiated air-liquid interface (ALI) cultures of human primary bronchial epithelial cells (PBEC) to study biofilm formation and their phenotypes by four mycobacterial species: Mtb*, M. bovis* (BCG), *M. avium* (Mav) and *M. smegmatis* (Msmeg). We delineated biofilm formation and adherence to PBEC over time and determined matrix biomass and polysaccharide accumulation. Finally, we investigated the effects of biofilms on PBEC barrier function and cell viability.

## Results

### Mycobacteria form biofilms on plastic

**Figure 1:**
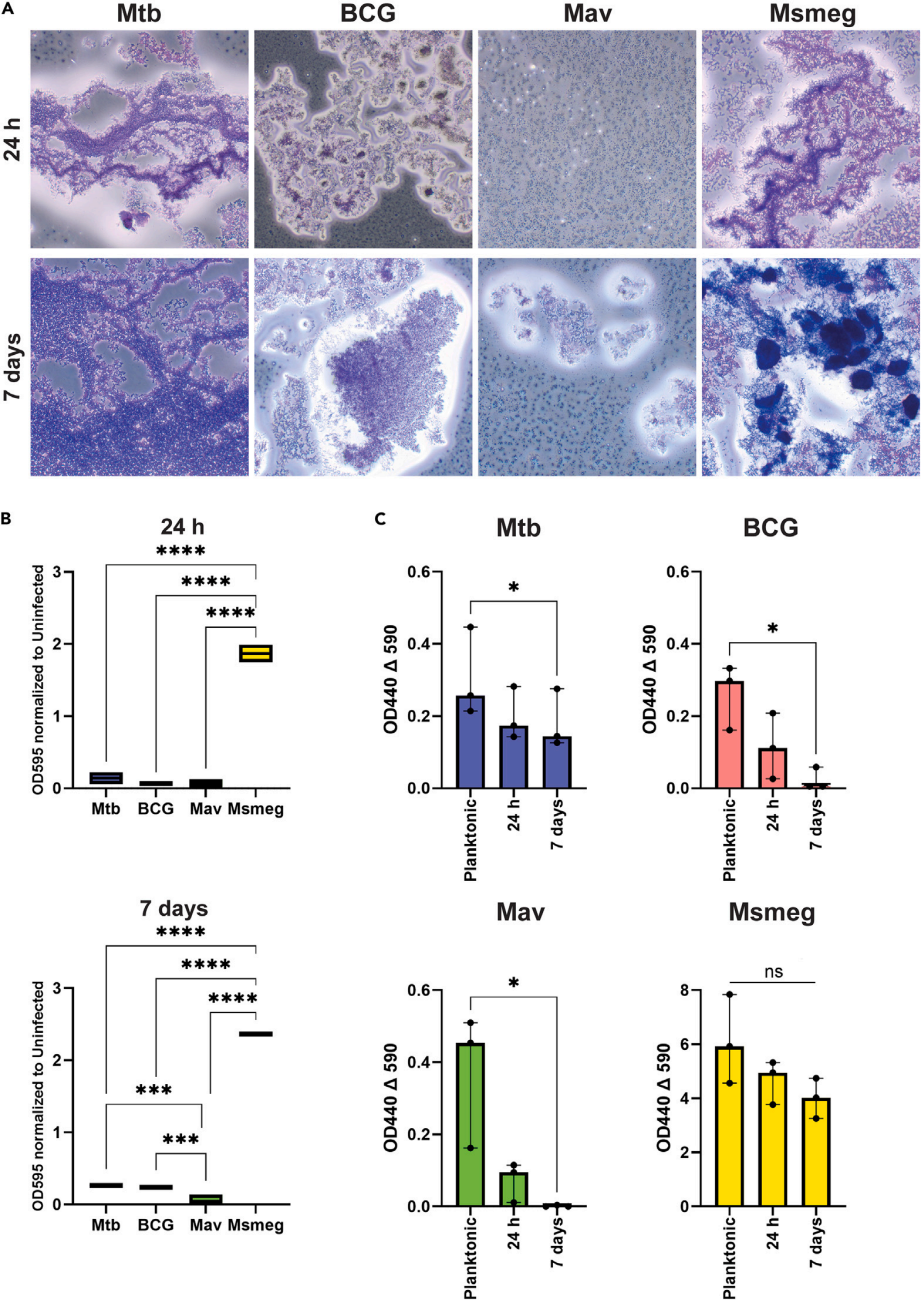
Formation and metabolic activity of mycobacterial aggregates attached to abiotic surfaces (A) Light microscopy images of 1x10^8^ bacteria cultured for 24 h or 7 days on tissue culture plates, and stained with crystal violet. Representative images from *N* = 3 independent experiments. (B) Boxplots show the optical density of crystal violet stained bacterial cultures. *N* = 2 independent experiments with 4–5 technical replicates (single biofilms) per experiment. Statistics performed as two-way ANOVA with Tukey correction for multiple testing. (C) Bar graphs show the metabolic activity of planktonic bacteria and biofilms grown on tissue culture plates. *N* = 3 independent experiments. Data are shown as median with range. Statistics were performed as the Kruskal-Wallis test with Dunn’s correction for multiple testing. *p* < 0.05 indicated with ∗, *p* < 0.01 ∗∗, *p* < 0.001 ∗∗∗, *p* < 0.0001 ∗∗∗∗.

We first investigated the capacity of mycobacteria to form biofilms on an abiotic plastic surface. Mtb, BCG, Mav, and Msmeg were incubated on plastic cell culture plates at a density of 1x10^8^ bacteria for 24 h and 7 days. In these time frames, we expected mycobacteria to form less developed and more developed biofilms, respectively. After incubation, biofilms were washed several times to remove non-adherent bacteria prior to performing subsequent assays. After 24 h, all four mycobacterial species adhered to the plastic (Figure 1A). Interestingly, the morphology of Mtb, BCG, and Msmeg aggregations was more cluster- or cord-like, whereas Mav covered the plastic in a more homogeneous manner. After 7 days, the appearance of bacterial aggregations was largely similar to those at 24 h. Msmeg bacilli had grown to a noticeably higher density at this time point, and Mav displayed a cluster-like morphology in some areas (Figure 1A).

For these structures to qualify as biofilms, bacteria should secrete an extracellular matrix that can be detected using crystal violet staining (CV). CV stains both the bacteria that adhere to a surface as well as any matrix they secrete, while non-adherent bacteria are rinsed away during the procedure, thereby making the total biofilm biomass quantifiable. Msmeg produced the highest biofilm biomass at both 24 h and 7 days (Figure 1B), which could be partly due to its higher growth rate compared to the other species. The other species produced biofilms with a 10-fold lower biomass at both time points. Msmeg was able to form biofilms in both epithelial cell culture medium and 7H9 broth (Figure S1), while the other species could not form biofilms in 7H9, though they did in cBD medium.

To compare the metabolic activity of planktonic mycobacteria to those in biofilms, conversion of tetrazolium salts to formazans was measured. In the planktonic state, the slow-growing Mtb, BCG, and Mav displayed comparable metabolic activity, which was significantly reduced after 7 days of biofilm formation (Figure 1C). As expected, fast-growing Msmeg had an over 10-fold higher metabolic activity than the other species in the planktonic state, and this activity was not significantly reduced upon biofilm formation. Interestingly, the metabolic activity of Mtb was reduced at day 7 compared to planktonic, but the magnitude was less compared to BCG and Mav.

### Mycobacteria form biofilms on bronchial epithelial cells

**Figure 2:**
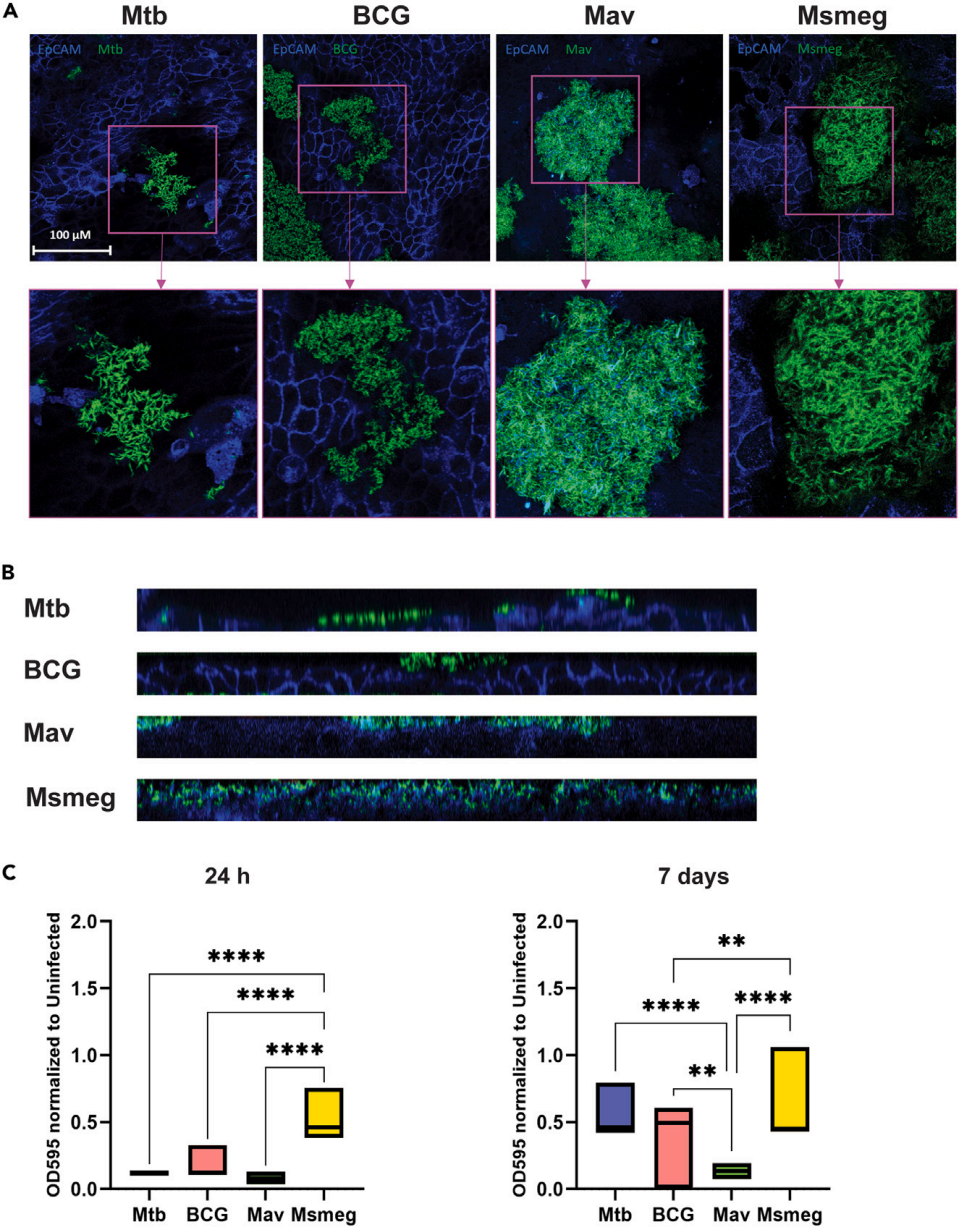
Mycobacterial aggregations are formed on bronchial epithelial cells (A) Confocal microscopy images of primary bronchial epithelial cells (PBEC) cultured at an air-liquid interface for 14 days, infected with bacteria at a multiplicity of infection (MOI) of 100, taken at 24 h post-infection. EpCAM membrane marker is depicted in blue, bacteria are depicted in green. Representative images from *n* = 3 independent experiments. (B) Side views of z-stacks of 24 h bacterial infections depicted in A (total images, not cut-outs). (C) Boxplots show the optical density of crystal violet stained bacteria cultured on well-differentiated PBEC. *N* = 3 independent experiments using 3 different donor mixes, with 5 technical replicates (single PBEC inserts) per experiment. Statistics were performed as two-way ANOVA with Tukey’s correction for multiple testing. *p* < 0.05 indicated with ∗, *p* < 0.01 ∗∗, *p* < 0.001 ∗∗∗, *p* < 0.0001 ∗∗∗∗.

To determine if mycobacteria could form biofilms on a relevant biotic surface such as bronchial epithelial cells, PBEC cultures consisting of four pooled donors each were infected for 24 h after which bacterial aggregation was evaluated by confocal microscopy. All species formed bacterial clusters on PBEC (Figure 2A). These clusters varied in size and were found in various locations on the PBEC cell layer, but did not cover the entire surface of the cell layer. Furthermore, the clusters of Msmeg seemed to partially penetrate the cell layer (Figure 2B), and clusters often co-localized with cell debris, indicating damage to the epithelial cell layer.

To confirm bacterial adherence to the PBEC cultures and the presence of an extracellular matrix, co-cultures of 24 h and 7 days were established and subsequently stained with crystal violet. After 24 h, Msmeg biofilms had the highest biomass which correlated well with our observations on plastic surfaces, although the total biomass was lower on PBEC than on plastic at both time points (Figure 2C). At 7 days, Mtb and BCG biofilms on PBEC reached a similar biomass as Msmeg. Mav did not form biofilms with a high biomass on PBEC at either time point.

**Figure 3:**
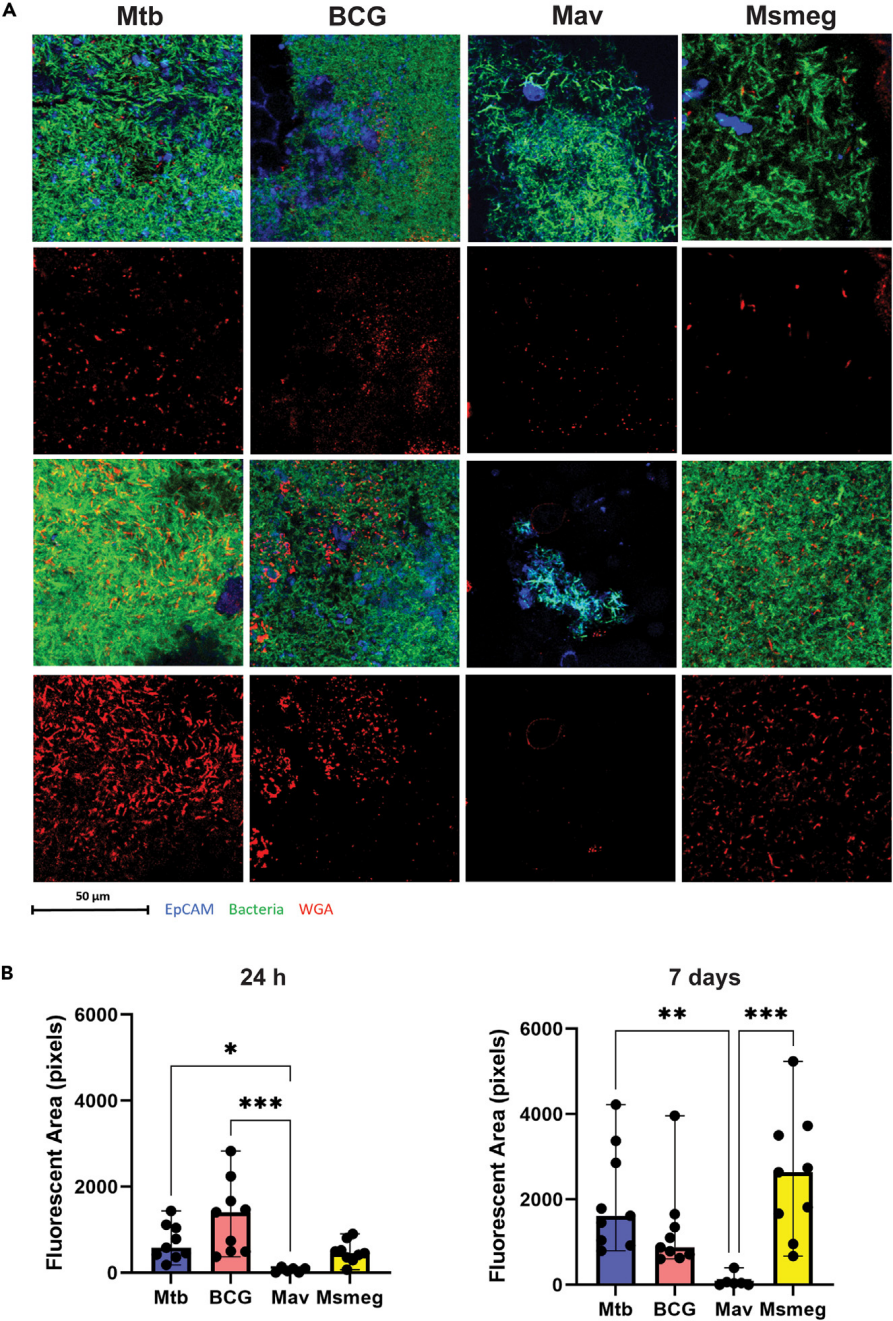
Polysaccharides secreted by mycobacteria increase as biofilms develop (A) Confocal microscopy images of 24 h and 7-day mycobacterial infections cultured on well-differentiated PBEC, stained with wheat germ agglutinin (WGA) to indicate polysaccharides. EpCAM is depicted in blue, bacteria in green, and WGA in red. Representative images from *N* = 3 independent experiments using three different donor mixes. (B) Bar graphs depicting the total area in pixels which was positive for WGA staining. Per PBEC insert, one 50 × 50 μm region of interest within the biofilm was selected for analysis. Data are shown as median with range. Statistics performed as Friedman test with Dunn’s correction for multiple testing. *p* < 0.05 indicated with ∗, *p* < 0.01 ∗∗, *p* < 0.001 ∗∗∗.

Previous reports have suggested cellulose, a polysaccharide, as a main component of mycobacterial biofilm matrix.^12^ To confirm that the matrix formed by our strains also contained polysaccharides, a wheat germ agglutinin (WGA) staining was performed (Figure 3A). Biofilm-associated polysaccharides were detected in PBEC cultures with both 24 h and 7-day biofilms of Mtb, BCG, and Msmeg, and they co-localized with bacterial clusters. Polysaccharides were mostly absent in Mav-infected PBEC cultures. At 7 days, an increase of polysaccharides was observed in Mtb and Msmeg cultures compared to 24 h cultures, while BCG and Mav 7-day cultures contained approximately the same amount as 24 h cultures. These observations were confirmed by the quantification of the area positive for WGA staining (Figure 3B). In addition, Mav-infected cultures contained significantly less polysaccharides than the other species at both time points, which aligns well with our observation that Mav does not produce biofilms with high biomass.

Taken together, the aggregation of mycobacteria combined with the presence of an extracellular matrix indicates that Mtb, BCG, and Msmeg form biofilms on bronchial epithelial cells.

### Mycobacteria disrupt epithelial barrier integrity and function

**Figure 4:**
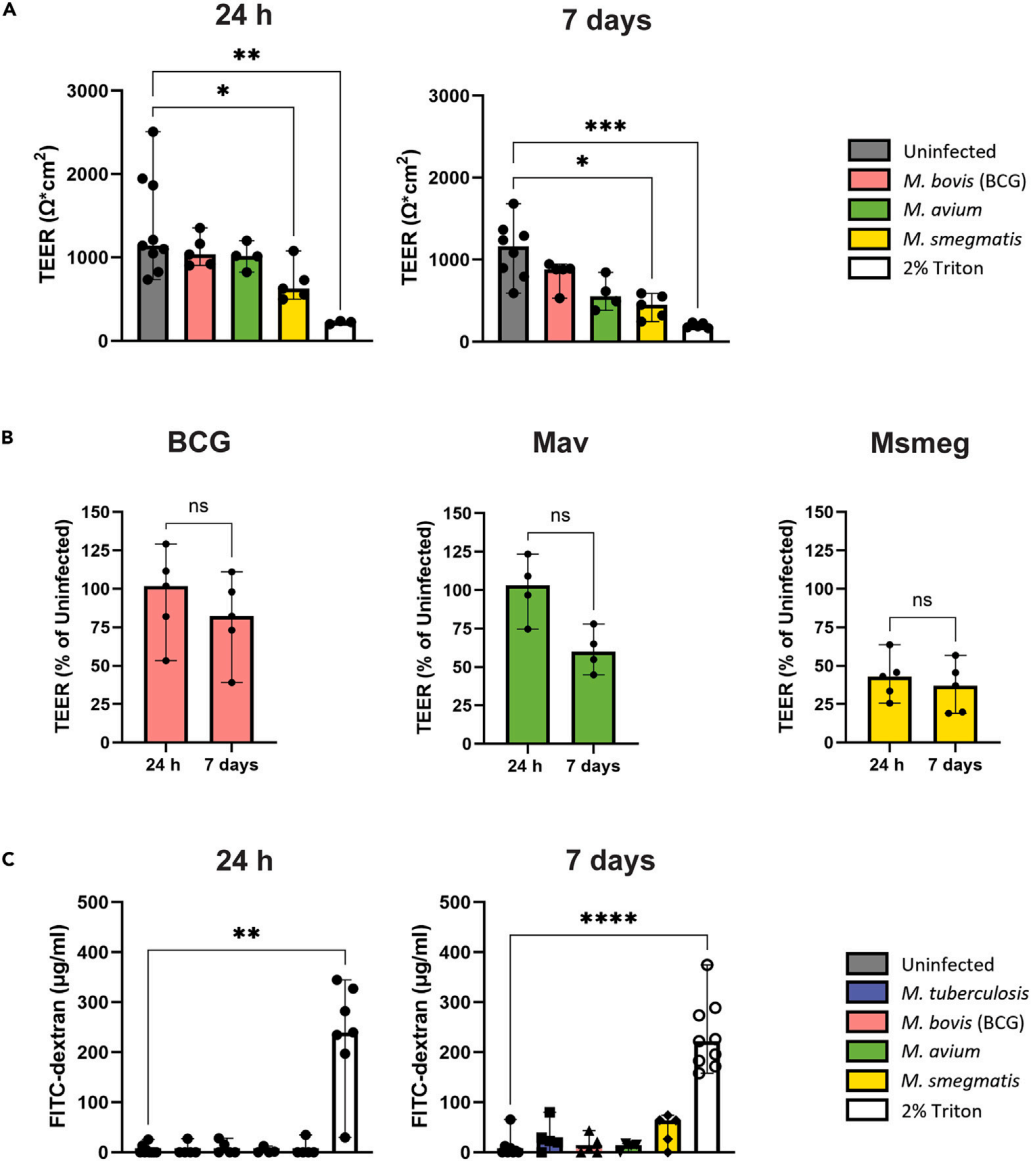
PBEC barrier integrity is disrupted by mycobacterial infection (A and B) Bar graphs of TEER measured on PBEC after 24 h and 7 days of biofilm formation. Positive control is 2% Triton X-100. N = at least 4 independent experiments. Data are shown as median with range. (C) Bar graph shows the passage of FITC-dextran through PBEC after 24 h and 7 days of biofilm formation. Positive control is 2% Triton X-100. N = at least 4 independent experiments. Data are shown as median with range. Statistics are performed as the Kruskal-Wallis test with Dunn’s correction for multiple testing. *p* < 0.05 indicated with ∗, *p* < 0.01 ∗∗, *p* < 0.001 ∗∗∗, *p* < 0.0001 ∗∗∗∗.

Epithelial barrier integrity after mycobacterial infection was investigated, as this integrity is crucial to maintaining a healthy mucosal barrier. Barrier integrity was affected post-infection with BCG, Mav, and Msmeg as demonstrated by reduced transepithelial electrical resistance (TEER), particularly after 7 days (Figure 4A). Due to BSL-3 restrictions, the TEER of Mtb-infected PBEC could not be measured. Compared to uninfected PBEC, Msmeg most drastically reduced TEER at both time points, followed by Mav. Msmeg reduced TEER to a similar extent at 7 days compared to 24 h*,* while BCG and Mav reduced TEER more strongly at 7 days than 24 h, although this difference was not statistically significant (Figure 4B). In addition, TEER measurements were variable between different donor pools, as some had a more drastic reduction in TEER between 24 h and 7 days post-infection, while others remained stable (Figure S2A), indicating that donor variability could play a role in how strongly epithelial cells react to mycobacteria.

Epithelial cells form a tight barrier that will not allow passage of large molecules. To determine whether mycobacterial infection affected this barrier function, the passage of 4 kDa FITC-dextran through the infected PBEC cell layer was measured. After 24 h of infection, there was barely any passage of FITC-dextran (Figures 4C and S2B). However, after 7 days, all species damaged PBEC to allow passage of FITC-dextran to variable extent. Msmeg-infected PBEC leaked the highest amount of FITC-dextran through the cell layer, followed by Mtb, albeit still relatively little compared to the positive control. To summarize, we show that mycobacterial infection is detrimental to the epithelial barrier integrity and function of the host, particularly upon longer exposure.

### Mycobacteria preferentially induce non-apoptotic cell death in bronchial epithelial cells

**Figure 5:**
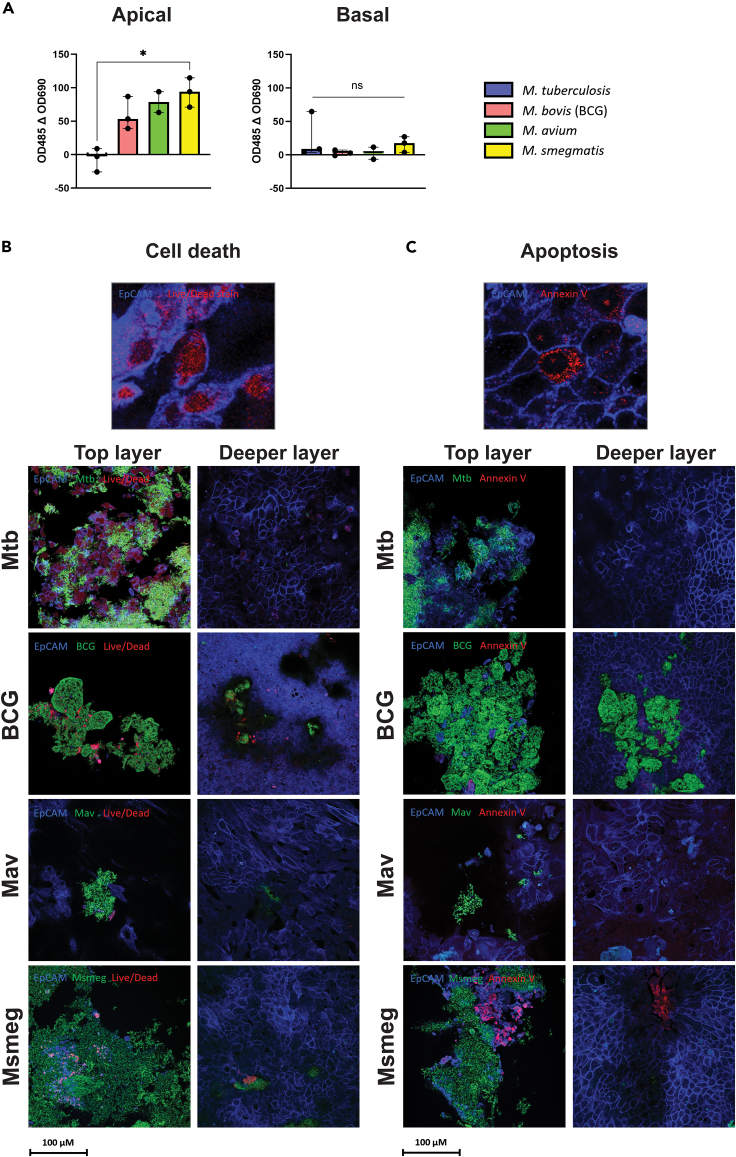
Cell death is induced upon contact with mycobacterial (A) Bar graphs showing percentage cytotoxicity induced by the presence of mycobacteria on PBEC. Percentages were calculated from the LDH release (see STAR Methods section). Data are shown as median with range. Statistics are performed as the Kruskal-Wallis test with Dunn’s correction for multiple testing. *p* < 0.05 indicated with ∗, *p* < 0.01 ∗∗, *p* < 0.001 ∗∗∗, *p* < 0.0001 ∗∗∗∗. (B and C) Confocal microscopy images of cell death (B) and apoptosis (C) induced by mycobacteria at 24 h, at the point of contact between biofilms and PBEC (top layer) and in a deeper cell layer of the same PBEC insert. Top panels are zoomed in examples of cell death and apoptosis staining in uninfected PBEC. Images from *N* = 2 independent experiments. EpCAM is depicted in blue, bacteria in green, and cell death markers in red.

Cytotoxicity of mycobacteria to PBEC cultures was measured via a lactate dehydrogenase (LDH) release assay after 24 h of infection. In the basal compartment, LDH levels were relatively low (Figure 5A). However, analysis of apical washes revealed high cytotoxic activity for all species except Mtb, with Mav and Msmeg inducing the highest release of LDH. Mycobacteria themselves did not secrete significant amounts of LDH (Figure S3).

Annexin V staining and a general cell viability dye were used to indicate apoptosis and other types of cell death, respectively. As is evident from the absence of annexin V staining, cells infected with Mtb, BCG, and Mav died via non-apoptotic cell death (Figures 5B and 5C). Cells in cultures infected with Msmeg, however, died via both apoptosis and other (unknown) mechanisms. Mostly, cells that were directly in contact with the bacteria were positive for cell death staining. Cell death staining in deeper layers, which were not in contact with bacteria, was absent or greatly reduced, which corresponds well with the LDH release data and indicates that the induction of cell death is likely contact-dependent.

### Morphology and formation of mycobacterial biofilms over time

**Figure 6:**
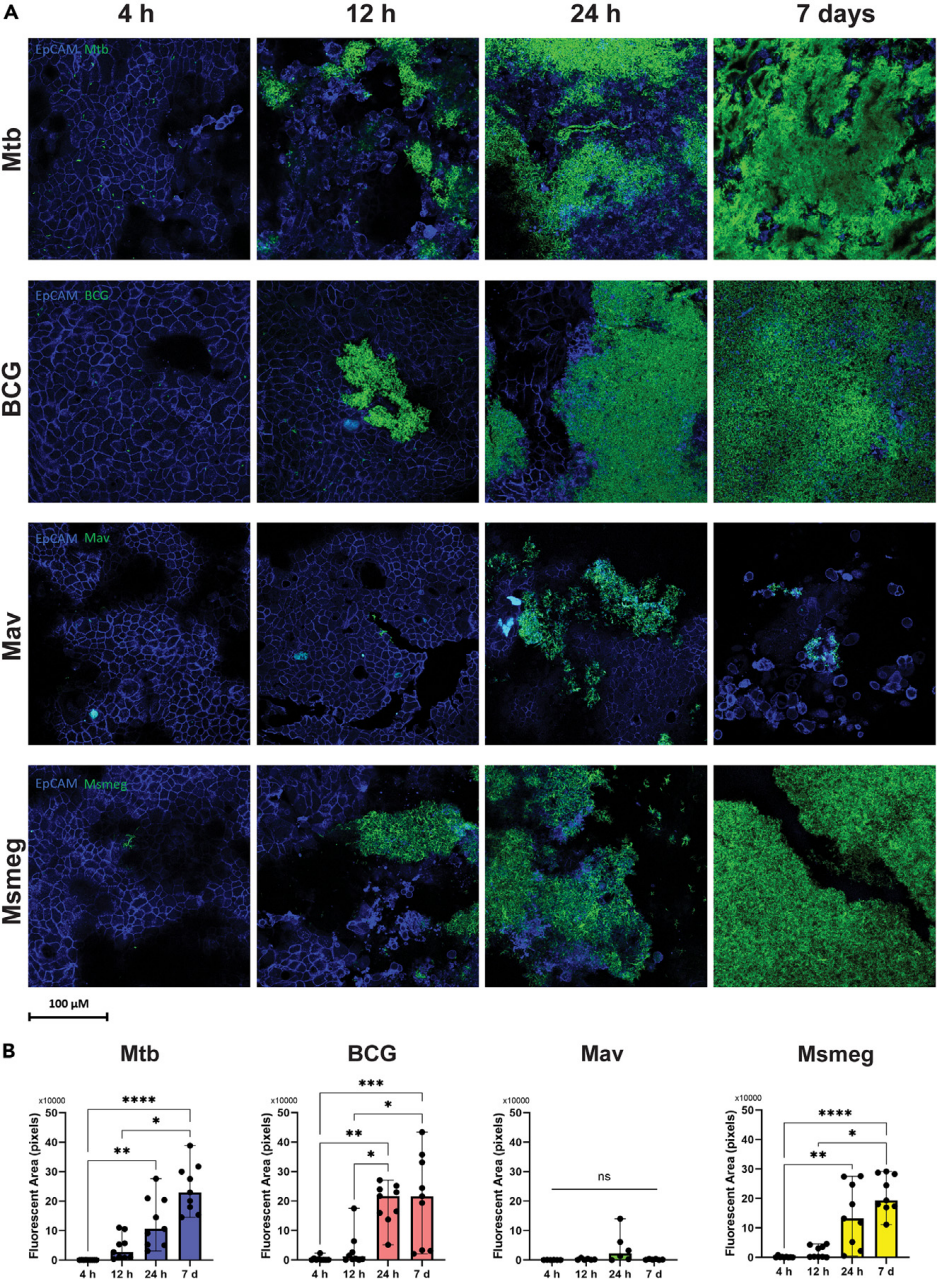
Mycobacterial biofilms are formed within 24 h of infection (A) Confocal microscopy images of the formation of biofilms and secretion of polysaccharides at 4 h, 12 h, 24 h, and 7 days. Images from N = at least 2 independent experiments. EpCAM is depicted in blue, and bacteria in green. (B) Bar graphs depicting the total area in pixels positive for fluorescent bacteria. Data are shown as median with range. Data points are single-plane images of z-stacks. Per the experiment, the 3 planes with the largest area of bacteria were chosen for quantification. Statistics performed as Friedman test with Dunn’s correction for multiple testing. *p* < 0.05 indicated with ∗, *p* < 0.01 ∗∗, *p* < 0.001 ∗∗∗, *p* < 0.0001 ∗∗∗∗.

The formation of mycobacterial biofilms on bronchial epithelial cells was followed over a time span of 4 h, 12 h, 24 h, and 7 days (Figure 6A). Biofilm formation occurred in approximately the same time frame for the mycobacteria tested. After 4 h of infection, few bacteria had adhered to the PBEC cell layer and no aggregation of bacteria was observed. At 12 h, small clusters of bacteria of each species were detected in several locations on the cell layer, and at 24 h, larger clusters of bacteria were observed. At 7 days, large parts of the epithelial cell layer were covered by a layer of bacilli, with the exception of Mav clusters which remained small. Quantification of the bacterial fluorescence showed an increase over time (Figure 6B).

Mucociliary clearance is an important process performed by airway epithelial cells to remove mucus-trapped foreign substances in the lung. We therefore hypothesized that mucus may hamper biofilm formation. Biofilms of mycobacteria were grown on plastic in a mixture of epithelial cell culture medium and mucus collected from uninfected PBEC. At 24 h, biofilms grown in the presence of mucus appeared to have a slight reduction in biomass compared to biofilms grown without mucus, though this was not statistically significant (Figure S4A). At 7 days this effect of the mucus was abrogated. Next, the effect of ciliated cells on BCG biofilm formation was assessed using PBEC that were air-exposed for 5 weeks instead of 2 weeks, which was previously shown to result in a higher percentage of ciliated cells in the culture.^22^ BCG biofilms formed in the same time frame on PBEC that were air exposed for 2 and 5 weeks (Figures S4B and S4C). Thus, it is unlikely that improved mucociliary function could prevent biofilm formation in our *in vitro* model.

**Figure 7:**
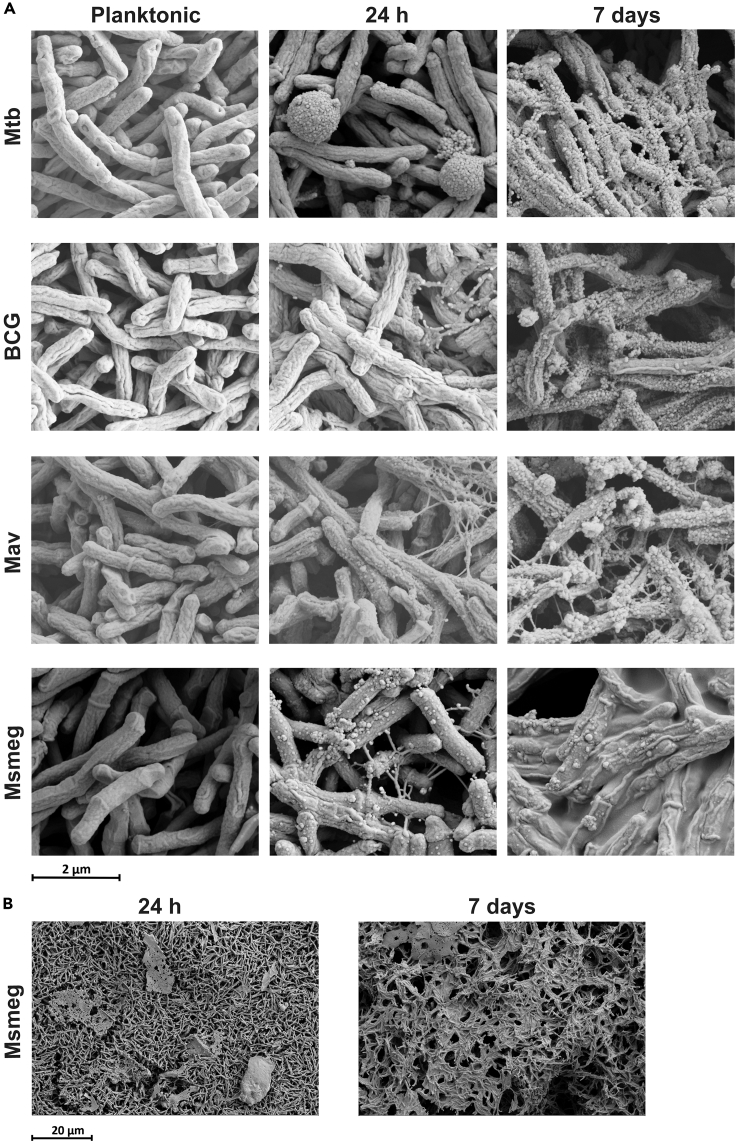
Morphological differences between planktonic and biofilm-associated mycobacteria (A) Scanning electron microscopy images of mycobacteria from liquid culture (planktonic) and biofilms of 24 h and 7 days grown on PBEC. Images from *N* = 1 experiment, taken at 150,000× magnification. (B) Scanning electron microscopy images of 24 h and 7-day Msmeg biofilms on PBEC show the formation of pore-like structures. Images taken at 10.000× magnification.

To assess the structure of the biofilm matrix and mycobacterial morphology, 24 h and 7-day biofilms on PBEC as well as planktonic mycobacterial cultures were examined by scanning electron microscopy (Figure 7A). Mycobacteria in the planktonic state had a relatively smooth appearance, and all species displayed the typical rod morphology. Mycobacteria in 24 h biofilms presented with a rougher texture compared to planktonic bacteria, as small specks and threads of biofilm matrix appeared on and in between bacteria. Interestingly, besides rod-shaped bacteria, ball-shaped forms were present in Mtb biofilms (Figure S5). In 7-day biofilms, matrix aggregation increased further, covering the majority of bacteria in the biofilm (Figure 7B). Over time, biofilms of some species also appeared to become more organized. At 24 h, bacteria in Mtb and Msmeg biofilms were randomly oriented, whereas at 7 days these biofilms had formed pore-like structures (Figures 7B and S6).

## Discussion

Tuberculosis and other mycobacterial infections share several properties with biofilm-associated infections, and biofilms may be of concern particularly for patients with active disease. Here, we show biofilm formation on plastic and primary bronchial epithelial cells by four mycobacterial species: *M. tuberculosis*, *M. bovis* (BCG), *M. avium,* and *M. smegmatis.* Biofilms formed by these bacteria had extracellular matrixes containing polysaccharides, though biomass and polysaccharide content varied by species. Mtb, BCG, and Msmeg were able to adhere to PBEC and consistently formed biofilms within 24 h, and most biofilms developed further over 7 days, increasing in biomass. Furthermore, the infection of bronchial epithelial cultures by mycobacteria appeared to be detrimental to host cells and was accompanied by loss of epithelial barrier integrity and function, and by the induction of mostly non-apoptotic cell death.

Though biofilms were formed by all four mycobacterial species, biomass and polysaccharide content varied between species. Mav produced the least amount of polysaccharides, and had a lower biofilm biomass than other species at 7 days. This was the case both on PBEC and plastic, indicating it is likely species or strain-dependent. It should be noted that other strains of Mav can form biofilms,^15^^,^^18^ and that their capacity to form biofilms is not dependent on smooth or rough colony morphology.^15^ Compared to our Mav strain, Mtb formed biofilms with high biomass and polysaccharide content. We have previously shown that out of these four species, Mav is most efficient at infecting PBEC intracellularly and Mtb is the least efficient, though it should be noted that in our previous study, the infection lasted 48 h rather than the 24 h or 7-day time points used in this study.^21^ The difference in infection efficiency might suggest that biofilm formation negatively impacts a species’ capacity to infect host cells, or that biofilm formation is a consequence of relatively low infection efficacy. Such a phenomenon has also been described for *Salmonella enterica.* In a mouse model, *S. enterica* serovar Typhimurium mutants that did not express extracellular matrix components invaded macrophages more efficiently than wild-type bacteria that formed biofilms.^23^ In competitive infection experiments, non-biofilm-forming *S. enterica* consistently outcompeted biofilm-forming bacteria.^24^^,^^25^ Therefore, biofilm formation may be a mechanism for bacterial survival when host cell infection is less successful. However, the opposite has been described for Mav complex strain A5 in the human bronchial epithelial cell line BEAS-2B. In this model, biofilm-deficient transposon mutants showed impaired invasion of the cells.^18^ More evidence suggesting that biofilm formation could be favorable for host cell invasion comes from a recent study by Mishra et al., who suggested that the cording of Mtb, which could be considered an early stage of biofilm formation, may assist in the dissemination of the bacteria throughout the lung.^10^ Whether biofilm formation hampers or expedites epithelial cell invasion remains to be further investigated.

Metabolic activity of mycobacteria differed per species and between planktonic and biofilm-associated bacteria. Of note, the metabolic activity of Mtb in biofilms remained comparable to planktonic Mtb, whereas the activity of other species declined markedly once they reside in biofilms. This phenomenon has been described previously by Trivedi et al. who demonstrated that reductive stress induced biofilm formation in Mtb shaking flask cultures, and that these biofilms harbored metabolically active bacteria that were tolerant to rifampicin and ethambutol.^26^ This suggests a mechanism other than the formation of a persister phenotype, as such bacteria are typically metabolically repressed.

Exposure of PBEC to mycobacteria resulted in barrier disruption and cell death, although we could not link this solely to biofilm formation. Particularly the non-tuberculous Msmeg was highly cytotoxic and disruptive compared to the other three species. This barrier disruption by BCG, Mav, and Msmeg is in line with previous findings that Mtb reduces TEER of rat alveolar epithelium, which was shown to be mediated via TNFα.^27^ However, this study used planktonic Mtb rather than biofilms. This indicates that the disruption of epithelial barriers by mycobacteria may not be exclusively linked to biofilms, but could be a consequence of exposure to mycobacteria. Furthermore, in this study, we could not differentiate between cell death induced by bacteria or by secreted virulence factors. Interestingly, Mtb, BCG, and Mav all induced non-apoptotic cell death, whereas Msmeg also induced apoptosis. A previous study comparing infections with the facultative-pathogens BCG and *M. kansasii*, and the non-pathogenic Msmeg in the human THP-1 monocytic cell line and murine bone marrow-derived macrophages found that only Msmeg induced apoptosis.^28^ Induction of apoptosis might be considered a host-protective mechanism, as bacteria are contained within apoptotic vesicles, preventing them from disseminating extracellularly.

For most of the species tested, a polysaccharide-containing matrix suggestive of biofilm formation was formed within 24 h. When comparing confocal data with data obtained by SEM, there was noticeably less matrix visible in samples prepared for SEM. This could be a result of the additional washing and processing steps required for this technique. Alternatively, the resolution of our SEM images might not be high enough to detect single polysaccharides, whereas those could be fluorescently labeled and imaged by confocal microscopy.

Morphological analysis of Mtb biofilms revealed ball-shaped forms in 24 h and 7-day biofilms. These appeared to be attached to, or perhaps emerge from, rod-shaped bacteria via thread-like structures. This led us to hypothesize that these ball-shaped forms may be cell wall-deficient (CWD) bacilli. A recent study by Dannenberg et al. provided compelling evidence that Mtb, BCG, and *M. marinum* are capable of shedding their cell wall in response to environmental stressors.^29^ These CWD forms were shown to be viable and replication competent, but not detectable by conventional TB diagnostic tools. Of note, they demonstrated that the cell wall-targeting antibiotics vancomycin and isoniazid stimulate the formation of CWD forms in BCG and Msmeg strains. Another study found CWD Mtb to be highly tolerant to ethambutol, in the absence of genetic resistance mutations.^30^ Moreover, shedding their cell wall might be advantageous for mycobacteria to subvert the immune system, as many important mycobacterial pattern recognition receptor agonists as well as antigens are cell wall-associated lipoproteins and glycolipids.^31^ The granuloma environment is hypoxic, and Mtb adapts to this environment by decreasing metabolic activity. It has been suggested that Mtb may survive within granulomas as CWD bacteria,^32^ although another study has shown that Mtb developed a thickened cell wall in hypoxic conditions.^33^ Based on the above morphological observations, we hypothesize that Mtb possibly developed cell wall-deficient forms in biofilms on PBEC, a phenomenon that has not been previously described. CWD Mtb may be involved in bacterial persistence, and their role in TB pathogenesis merits further investigation.

Another interesting structural aspect of mycobacterial biofilms that we observed is the increased degree of organization in 7-day biofilms compared to 24 h biofilms. At 7 days, pore-like holes or channels appeared to form. This is especially apparent in Msmeg biofilms, where these holes are clearly visible in the biofilm matrix. As only small representative parts of each sample were imaged, reliable quantification of these structures was not possible. In biofilms of other bacterial species, channels, and pores serve to transport or store nutrients and waste products.^34^ The presence of such pores and channels was previously identified in Mtb biofilms grown in shaking liquid cultures.^26^ Networks of well-defined channels have also been found in *Bacillus subtilis* biofilms, which provide effective transport of liquids through the biofilm.^35^ Thus, the poriferous structures we observed may contribute to nutrient and waste product transport in mycobacterial biofilms.

Biofilms are generally considered detrimental to human and animal health. In patients with CF , as mentioned, *P. aeruginosa* biofilms contribute to chronic infection and their matrixes contain polysaccharides that interfere with antibiotic and anti-biofilm treatments.^36^ Biofilms of *S. aureus* growing on orthopedic implant material are notoriously difficult to eradicate and may harbor persisters that require alternative treatment strategies.^37^ Thus, biofilm formation may also have implications for the clinical treatment of TB, particularly in the case of recurrent infections and multi-drug resistant Mtb.

Relapse of TB occurs in a proportion of patients, and drug-resistant Mtb strains may arise in these patients. Drug resistance may be due to acquired genetic mutations. However, host-derived stresses on the bacteria may also induce physiological changes that render them recalcitrant to antibiotic therapy. For example, Mtb isolated from infected mice exhibits tolerance to multiple frontline drugs to a degree proportional to the activation status of the host immune cells.^38^ Another strategy to achieve drug tolerance in many bacterial species is biofilm formation. Ackart et al. showed that Mtb growing as attached microbial communities *in vitro* were significantly less susceptible to rifampicin and isoniazid than planktonic Mtb.^39^ In guinea pigs infected with Mtb, which form lung lesions with high similarity to humans, drug-tolerant persisting bacteria were found in the acellular rim of necrotizing lesions.^40^ However, there is currently no definitive answer to the question of whether mycobacteria in necrotizing granulomas can truly be considered biofilms. Nevertheless, understanding mycobacterial biofilm formation in the lung might assist in improving treatment strategies. For example, a study using an Mtb *Δmtp* mutant strain incapable of forming pili showed that the biofilm mass produced by this strain was reduced by over 68% compared to wildtype Mtb,^41^ marking pili as an anti-biofilm therapeutic target.

In conclusion, we have demonstrated that Mtb and three other species of mycobacteria formed organized biofilms on cultures of well-differentiated primary human bronchial epithelial cells, with several species disrupting host barrier function and causing cell death. In addition, we identified potentially cell wall-deficient Mtb within biofilms. This work advances our understanding of mycobacterial biofilm formation in the lung and its consequences for the mucosal barrier and provides a starting point for additional research into the role of biofilms in TB pathogenesis and drug tolerance.

### Limitations of study

A limitation of our study is the absence of immune cells and the natural lung microbiome. As the lung is colonized by many microbial species, it is unlikely that biofilms in the lungs of patients with TB only contain mycobacteria. The presence of other microbial species may influence TB pathogenesis and treatment outcome, as was suggested by a study comparing the microbiomes of patients recently diagnosed with TB, patients with recurrent TB, and patients with treatment failure.^42^ For example, these studies showed that *Pseudomonas* species are more abundant in the lung microbiome of patients with TB who present with treatment failure compared to those with new or recurrent infections. This may be of particular relevance for patients with chronic obstructive pulmonary disease (COPD) or cystic fibrosis (CF), as *Pseudomonas* is a genus often associated with chronic infections in these patients,^43^ and they have a greater propensity to contract TB than healthy individuals.^44^ Furthermore, the spatial organization of microbes within multi-species biofilms may lead to increased biomass and enhanced antibiotic tolerance compared to biofilms with only one species.^45^^,^^46^ Though current evidence from a limited number of studies suggests that the lung microbiome is associated with TB infection and disease severity, additional studies will be needed to determine clinical relevance.

Another limitation of this study is the high bacterial load of 1 × 10^8^ CFU used to infect PBEC, as it is unknown whether this accurately mimics conditions in humans *in vivo.* In non-human primates such as rhesus macaques, bacterial loads per granuloma range from zero to 1 × 10^7^ CFU.^47^ In cynomolgus macaques, the bacterial load and fate of individual lesions also vary considerably within single hosts.^48^^,^^49^ This substantial heterogeneity should be taken into account, as we hypothesize that biofilm formation is more likely upon the cavitation of a granuloma with high bacterial load.

We studied the effect of mucociliary clearance on biofilm formation by BCG in the *in vitro* airway culture model. While improved mucociliary clearance, achieved via extended differentiation of the models, could not perturb biofilm formation, it should be noted that mucus could not be removed from our *in vitro* model by ciliary beating, as is the case *in vivo*. This limits the utility of this model for studying mucociliary clearance.

## Resource availability

### Lead contact

Further information and requests for resources and reagents should be directed to and will be fulfilled by the lead contact, Simone Joosten (s.a.joosten@lumc.nl).

### Materials availability

This study did not generate new unique reagents.

### Data and code availability

• Crystal violet, LDH, WST, TEER, FITC-dextran, and microscopy quantification data have been deposited at Mendeley Data and are publicly available as of the date of publication. DOIs are listed in the key resources table. Raw confocal microscopy data reported in this article will be shared by the lead contact upon request.
• This article does not report the original code.
• Any additional information required to reanalyze the data reported in this article is available from the lead contact upon request.

## Acknowledgments

We gratefully acknowledge Prof. Dr. William R. Jacobs (Albert Einstein College of Medicine, Yeshiva University, New York City, United States) for kindly providing the Mtb strain constitutively expressing Venus. This work was supported by a grant from the Leiden-Edinburgh Joint PhD program for Integrated One Health Solutions, awarded by the universities of Leiden and Edinburgh in December 2019.

## Author contributions

AB collected and analyzed data and drafted the article. DN performed all cell culture work to create the PBEC *in vitro* models. RL performed all sample preparation and imaging for the scanning electron microscopy. KW performed TEER and FITC-dextran assays. MB, PH, TO, AD, and SJ served as scientific advisors and critically reviewed the study design and article.

## Declaration of interests

The authors declare no competing interests.

## STAR★Methods

### Key resources table

**Table:** 

REAGENT or RESOURCE	SOURCE	IDENTIFIER
**Antibodies**		

Donkey anti-Goat IgG (H + L) Highly Cross-Adsorbed Secondary Antibody, Alexa Fluor™ Plus 405	Invitrogen	Cat#A48259; RRID:AB_2890272
Donkey anti-Mouse IgG (H + L) Highly Cross-Adsorbed Secondary Antibody, Alexa Fluor™ 647	Invitrogen	Cat#A-31571; RRID:AB_2762830
Annexin V Monoclonal Antibody (C13)	Invitrogen	Cat#MA5-41552; RRID:AB_2899035
Human EpCAM/TROP-1 Antibody	R&D Systems	Cat#AF960; RRID:AB_355745

**Bacterial and virus strains**		

*Mycobacterium avium*	Previously generated at own lab	ATCC700898 strain with pSMT3-Wasabi plasmid
*Mycobacterium bovis*	Previously generated at own lab	BCG P3 strain with pSMTeGFP plasmid
*Mycobacterium smegmatis*	Previously generated at own lab	mc2155 strain with pSMTeGFP plasmid
*Mycobacterium tuberculosis*	Jacobs Lab, Bronx NY, USA	H37Rv mc28120 strain with pYUB2133-Venus plasmid

**Biological samples**		

Primary bronchial epithelial cells	See methods section under experimental model and study participant details: bronchial epithelial cell culture	

**Chemicals, peptides, and recombinant proteins**		

LIVE/DEAD™ Fixable Near IR (780) Viability Kit, for 633 nm excitation	Invitrogen	Cat#L34994
Wheat Germ Agglutinin (WGA)	Invitrogen	Cat#W21404
Crystal Violet Solution	Sigma-Aldrich	Cat#V5265
Fluorescein isothiocyanate–dextran	Sigma-Aldrich	Cat#46944
ProLong™ Diamond Antifade Mountant	Invitrogen	Cat#P36965

**Critical commercial assays**		

Cytotoxicity Detection Kit^PLUS^ (LDH)	Roche	Cat#11644793001
Cell Proliferation Reagent WST-1	Roche	Cat#11644807001

**Deposited data**		

Data generated in this study	Mendeley Data	https://doi.org/10.17632/xvzh5y5dhg.1

**Software and algorithms**		

GraphPad	Prism	Version 9.3.1
ImageJ	Fiji software	
Leica Application Suite X (LAS X)	Leica	Version 5.0.2

### Experimental model and study participant details

#### Bacteria

Mycobacteria were cultured in Difco Middlebrook 7H9 medium (BD Biosciences, Franklin Lakes, NJ, USA) containing 0.05% Tween-80 (Sigma-Aldrich, St. Louis, MO, USA), 0.5% glycerol (Sigma-Aldrich), and Middlebrook ADC growth supplement (750 mM BSA +110 mM dextrose +130 μM catalase in H_2_O, produced in-house). A list of bacterial strains used and their origins can be found in Table S1. The *M. avium* strain expressing Wasabi, the *M. bovis* BCG strain expressing GFP, and the *M. smegmatis* strain expressing GFP were cultured with 100 μg/mL hygromycin B (Invitrogen, Thermo Fisher Scientific, Waltham, MA, USA). The *M. tuberculosis* H37Rv strain expressed Venus constitutively. The day before experiments were performed, BCG, *M. avium* and *M. smegmatis* were diluted to OD_600_ = 0.25, and Mtb was diluted to OD_600_ = 0.4 to ensure exponential growth of the bacteria.

#### Bronchial epithelial cell culture

Primary human bronchial epithelial cells (PBEC) were isolated from macroscopically normal lung tissue obtained from patients undergoing resection surgery for lung cancer at the Leiden University Medical Center, the Netherlands. Initially, lung tissue samples were enrolled in the biobank via a no-objection system for coded anonymous further use of such tissue (www.coreon.org). Since 01-09-2022, patients are enrolled in the biobank using written informed consent in accordance with local regulations from the LUMC biobank and with approval by the institutional medical ethical committee (B20.042/Ab/ab and B20.042/Kb/kb). Cells were cultured as previously described.^50^ In brief, PBEC (passage 1) of individual donors were expanded for 5 days in T75 culture flasks pre-coated with a mixture of 30 μg/mL Purecol (Advanced BioMatrix, Carlsbad, CA, USA), 5 μg/mL human fibronectin (PromoCell, Heidelberg, Germany) and 10 μg/mL BSA (Fraction V; Thermo Fisher Scientific, Carlsbad, CA, USA). Per experiment, mixes of 4 unique donors were prepared^22^: PBEC of 2 male and 2 female donors were mixed, averaging out potential sex-based biological differences. Cells were derived from patients without COPD and seeded at equal concentrations and at a high density of 160.000 cells on pre-coated 0.4 μm membrane pore size polyethylene terephthalate (PET) inserts (cellQART, Northeim, Germany) in 12-well plates to rapidly obtain confluent cell layers and prevent excessive growth of a certain donor in the mix. Cells were cultured in medium consisting of one part Bronchial Epithelial Cell Medium-basal (BEpiCM-b (ScienCell, Carlsbad, CA, USA)) and one part Dulbecco’s modified Eagle’s medium (DMEM (STEMCELL Technologies, Vancouver, Canada)) supplemented with Bronchial Epithelial Cell Growth Supplement (BEpiCGS; ScienCell), 12.5 mM HEPES (Gibco, Thermo Fisher Scientific), 100 U/ml penicillin, 100 μg/mL streptomycin (Gibco), 2 mM glutaMAX (Gibco), further referred to as complete BD-medium (cBD). In the submerged stage, cBD was supplemented with 1 nM EC-23 (Tocris, Bristol, UK). EC-23 is a light-stable analogue of retinoic acid and supports mucociliary differentiation.^50^^,^^51^ After reaching 100% confluence, apical medium was removed and cells were differentiated at the air-liquid interface (ALI) in cBD supplemented with 50 nM EC-23. Culture medium was changed 3 times per week during which the apical side of the culture was carefully washed with 200 μL warm PBS for 10 min at 37°C to wash away mucus and cell debris. After 14 days of mucociliary differentiation at ALI, all dominant cell-types were present and ciliary activity was observed. Cultures were subsequently used for experiments. For long-term differentiation, PBEC were cultured for 5 weeks at ALI. During that time, culture medium was also changed 3 times per week, and mucus washed away as described above.

### Method details

#### Infection of airway epithelial cell cultures & biofilm formation

PBEC were cultured in medium without antibiotics 2–4 days before infection experiments. Mtb Venus, BCG GFP, Mav Wasabi and Msmeg GFP were cultured as described above. Although the addition of 0.05% Tween-80 to bacterial cultures minimized bacterial aggregation, minor clumping cannot be completely ruled out. OD_600_ of visually homogeneous bacterial cultures was measured. A multiplicity of infection (MOI) of 100 was prepared accordingly in cBD containing 50 nM EC-23. For these calculations, PBEC cell density was estimated at 1x10^6^ cells per insert. Bacterial suspensions were plated on 7H10 agar to retrospectively determine the actual infection load. Mucus was washed from the apical surface of PBEC cultures with PBS. Immediately following mucus removal, 50 μL bacterial suspension was added to the apical side of the cells. Control cultures received 50 μL medium without bacteria. Next, cultures were centrifuged briefly at 300 x g for 2 min to spin bacteria down, and incubated at 37°C for 24 h to obtain a less developed biofilm or 7 days to obtain a more developed biofilm. During a 7-day infection, PBEC were supplied basally with 1 mL new medium every 3 days.

#### Biofilm formation on plastic

Mycobacteria were grown in Middlebrook 7H9 medium as described above, and OD_600_ of bacterial cultures was measured. For assays using a flat bottom 96-wells plate (Corning Costar), a concentration of 1 x 10^7^ colony forming units (CFU)/mL was prepared accordingly in either 7H9 or cBD containing 50 nM EC-23, and 100 μL bacterial suspension was added per well. For assays using a flat bottom 24-wells plate (Corning Costar), a suspension of 2 x 10^9^ CFU/mL was prepared and 50 μL bacterial suspension was added per well and spread equally over the plastic. Bacteria were incubated at 37°C for 24 h to obtain a less developed biofilm or 7 days to obtain a more developed biofilm.

#### Crystal violet assay

Bacterial suspensions cultured on Transwell inserts and in plastic 24-wells or 96-wells plates were discarded. PBEC and plastic wells were carefully washed with 200 μL PBS for 10 min at 37°C. From PBEC, this first PBS wash as well as the cBD they were cultured in were collected and used in LDH assays (see below). PBEC and plastic wells were washed again once with PBS. Adherent bacteria were stained with 200 μL 1% Crystal Violet staining (Sigma-Aldrich) for 10 min at room temperature (RT). PBEC and plastic wells were washed 4x with demi H_2_O. Next, stained biofilms were either imaged using an IX51 light microscope (Olympus), or 200 μL 96% ethanol (Supelco) was added for 10 min at RT to dissolve the crystal violet staining for quantification. From each biofilm, 100 μL liquid was collected and transferred to a flat bottom 96-wells plate. OD_595_ was measured on an Envision Multimode Plate Reader (PerkinElmer, Waltham, Massachusetts, USA). Per experiment, at least 4 technical replicates were measured per condition.

#### WST-1 metabolic activity assay

Suspensions of 2 x 10^9^ planktonic bacteria per mL were used to measure baseline metabolic activity as follows. Of each bacterial species, 50 μL suspension was added to a flat bottom 96-wells plate. This plate was centrifuged 10 min at 805 x g and supernatant was discarded. Bacteria were incubated 30 min with cell proliferation reagent WST-1 (Roche) diluted 1:10 in cBD with 50 nM EC-23, according to manufacturer’s instructions. Plates were centrifuged again, and 100 μL of each well was collected. Optical density was measured at 440 nm and 590 nm using an Envision Multimode Plate Reader (PerkinElmer). Biofilms were prepared from the same bacterial suspensions in 24-wells plates as described above. Planktonic bacteria were removed by washing biofilms twice in PBS. Then, metabolic activity was measured in the same manner as the planktonic bacterial suspensions. Three technical replicates per condition were performed within each individual experiment.

#### Confocal microscopy

Following infection, PBEC cultures were carefully washed with PBS on both sides to remove traces of culture medium. Inserts for cell death experiments were immediately stained with 100 μl Fixable Live/Dead dye (Invitrogen), diluted 1:400 in PBS, for 30 min at room temperature (RT) and then washed with PBS again. Then, all PBEC were fixed in 4% paraformaldehyde (PFA) for 24 h at 4°C. After fixation, cultures were washed and stored submerged in PBS at 4°C until staining. Cell culture inserts were blocked with PBS +1% BSA +0.3% Triton X-100 (Fluka Chemie, Buchs, Switzerland) (PBT) for 10 min at RT. Semi-permeable membranes were removed from plastic inserts with a scalpel. Membranes were incubated with primary antibodies (Table S2) overnight at 4°C. Membranes were washed 3 times with PBS before incubation with secondary antibodies (Table S2) and other stains for 2 h at 4°C. Membranes were washed three times in PBS and three times in demi H_2_O, then placed on a glass slide, and treated with ProLong Diamond Antifade mountant (Invitrogen, Thermo Fisher Scientific). All samples were imaged with an SP8-WLL confocal microscope (Leica, Wetzlar, Germany) at the Light and Electron Microscopy Facility of the Leiden University Medical Center in Leiden, Netherlands (https://www.lumc.nl/research/facilities/light-and-electron-microscopy-facility/services/). Image processing was performed in Leica Application Suite X (LAS X) software. Further quantification of WGA staining and bacterial fluorescence were performed in Fiji ImageJ. To quantify WGA staining, regions of interest (ROIs) of 50 × 50 μm were selected in each biofilm, so that they only include WGA secreted by bacteria and not epithelial cells stained by WGA. The relevant fluorescent channels containing WGA staining or bacteria were then converted to a binary state to differentiate between fluorescent and non-fluorescent pixels. Fluorescent areas were measured with a 3-pixel minimum threshold per area. Total fluorescent area was calculated by summing up pixel sizes of all areas per ROI.

#### Trans-epithelial electrical resistance

As a positive control for barrier disruption, an uninfected PBEC insert was treated with 2% Triton X-100 in PBS for 5 min at RT. The solution was then removed and 700 μL PBS was added to all inserts for 10 min at RT. *Trans*-epithelial electrical resistance (TEER) was measured for 5–10 s using a STX2 electrode from World Precision Instruments (Sarasota, USA), connected to an EVOM2 TEER meter (World Precision Instruments). TEER was calculated as: $TEER=\Omega \;x\;{cm}^{2}$. The surface area of the 12-wells plate inserts used was 1.13 cm^2^. After measuring TEER, PBS was removed from the inserts and PBEC were used for the FITC-Dextran assay (see below).

#### FITC-dextran assay

As a positive control for barrier disruption, an uninfected PBEC insert was treated with 2% Triton X-100 in PBS for 5 min at RT. PBEC were incubated apically with 500 μL FITC-dextran (Sigma-Aldrich) at a concentration of 1 mg/mL in cBD for 2 h at 37°C. Then, 100 μL medium was collected from the basal compartment of each well and filtered using a FiltrEX 96-wells filter plate (Corning Costar) with pore size 0.2 μm to remove any bacteria. Filtered medium was transferred to a black 96-wells plate (Corning Costar). A 1:2 dilution series of FITC-dextran was prepared in cBD and used as a standard curve. Fluorescence was measured using a SpectraMax i3 (Molecular Devices), at 490 nm excitation. The concentrations of FITC-dextran that passed the epithelial barrier were interpolated from the standard dilution series. Three technical replicates per condition were performed within each individual experiment.

#### LDH assay

PBS washes from the apical side and cell culture medium from the basal side of infected PBEC collected during the Crystal Violet assay (see above) were used to measure LDH release with the Cytotoxicity Detection Kit (LDH) from Roche (Basel, Switzerland). To create a positive control sample, 200 μL PBS +2% Triton X-100 was added to the apical side of two uninfected PBEC cultures for 5 min at RT. From the apical sides and basal sides 100 μL was collected. As a background control, 100 μL cBD and 100 μL PBS were used. Then, 100 μL from all samples was transferred to a flat bottom 96-wells plate and centrifuged 3 min at 805 x g to spin down any cells and bacteria in the samples. Supernatants were transferred to a new flat bottom 96-wells plate. Reaction mixture from the kit was prepared according to manufacturer’s instructions and 100 μL was immediately added to each sample. Samples were left to incubate 28–30 min in the dark at RT, and then optical density at 485 nm and 690 nm were measured on an Envision Multimode Plate Reader. The percentage cytotoxicity was calculated: $\text{Cytotoxicity}\;\left(\%\right)=\frac{Sample\;value-Background}{Positive\;control-Background}\;x\;100$.

#### Scanning electron microscopy (SEM)

PBEC exposed to mycobacteria for 24 h or 7 days were fixed in 4% PFA +2% glutaraldehyde (Electron Microscopy Sciences) in 0.1 M cacodylate buffer (pH 7.4) for at least 30 min at RT followed by 24 h at 4°C. Suspensions of 2 x 10^9^ bacteria/ml were prepared directly from liquid mycobacterial cultures, and fixed in 4% PFA +2% glutaraldehyde for at least 30 min at RT followed by 3 days at 4°C. After washing with 0.1 M cacodylate buffer (pH 7.4), the samples were dehydrated in graded ethanol series. The samples were then immersed in 50% hexamethyldisilazane (HMDS) (Carl Roth) in ethanol for 30 min, followed by 100% HMDS for 30 min. HMDS was removed and samples were air dried. Filters were mounted on SEM stubs, sputter coated with Au/Pd and imaged with a Zeiss Gemini 300 scanning electron microscope, operated at 5.0 kV.

### Quantification and statistical analysis

Data analysis was performed in Graphpad Prism version 9.3.1. Statistical differences in TEER, FITC-dextran passage, LDH assays and WST assays were tested with a Kruskal-Wallis test + Dunn correction for multiple comparisons. Differences in crystal violet assays were tested with two-way ANOVA + Tukey correction for multiple testing. Differences in bacterial or WGA fluorescent area were tested with a Friedman test + Dunn correction for multiple testing. Threshold for statistical significance used was *p* < 0.05. Significance is indicated in figures as follows: *p* < 0.05 ∗, *p* < 0.01 ∗∗, *p* < 0.001 ∗∗∗, *p* < 0.0001 ∗∗∗∗. Sample size is indicated in figure legends as N.

## Supplemental information

Document S1. Figures S1‒S6 and Tables S1 and S2
