# Supplementary material for: Mycobacteria develop biofilms on airway epithelial cells and promote mucosal barrier disruption
Source: iScience. 2024 Sep 27;27(11):111063. doi: 10.1016/j.isci.2024.111063 (PMC11536035; doi:10.1016/j.isci.2024.111063)
Supplement: Document S1. Figures S1‒S6 and Tables S1 and S2 [file mmc1.pdf]

iScience, Volume *27*

Supplemental information

Mycobacteria develop biofilms on airway epithelial cells and promote mucosal barrier disruption

Amy M. Barclay, Dennis K. Ninaber, Ronald W.A. L. Limpens, Kimberley V. Walburg, Montserrat Bárcena, Pieter S. Hiemstra, Tom H.M. Ottenhoff, Anne M. van der Does, and Simone A. Joosten

Supplementary materials

Table S1. List of bacterial species used in this study. Related to STAR Methods.

**Bacterial species**	**Strain**	**Colony morphology**	**Fluorescent tag**	**Plasmid**	**Source**
Mycobacterium avium	ATCC 700898	Smooth	Wasabi	pSMT3-Wasabi	Fluorescent strain generated at own lab
Mycobacterium bovis	BCG P3	Rough	GFP	pSMT3eGFP	Fluorescent strain generated at own lab
Mycobacterium smegmatis	mc^2155	Rough	GFP	pSMT3eGFP	Fluorescent strain generated at own lab
Mycobacterium tuberculosis	H37Rv mc^28120	Rough	Venus	pYUB2133-Venus	Jacobs Lab, Bronx NY USA

Table S2. Antibodies and stains used for confocal microscopy. Related to STAR Methods.

**Target or name**	**Marker for**	**Host species**	**Target species**	**Concentration**	**Source**	**Cat. nr.**
AlexaFluor 405	Secondary Ab	Donkey	Goat	1:200	Invitrogen	A48259
AlexaFluor 647	Secondary Ab	Donkey	Mouse	1:200	Invitrogen	A-31571
Annexin V	Apoptosis	Mouse	Human	20 µg/ml	Invitrogen	MA5-41552
EpCAM	Epithelial cell membrane	Goat	Human	10 µg/ml	R&D Systems	AF960
Live/Dead NIR stain	Dead cells	-	-	1:800	Invitrogen	L34994
Wheat Germ Agglutinin 633 stain	Polysaccharides	-	-	5 µg/ml	Invitrogen	W21404

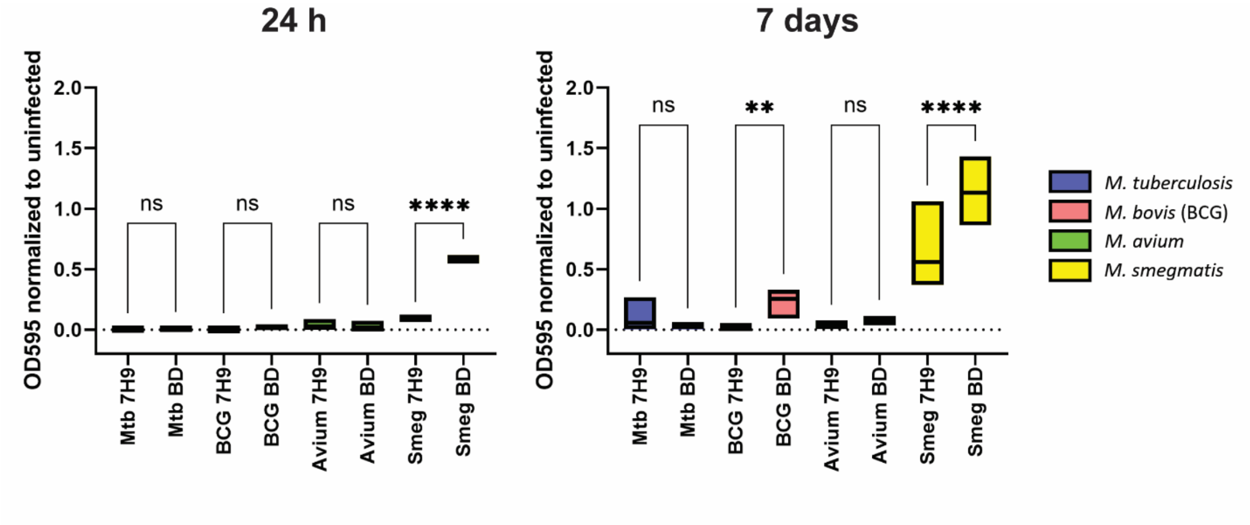

Figure S1. Mycobacterial biofilm formation in 7H9 and epithelial cell culture medium on an abiotic surface

Related to figure 1. Box plots showing optical density of crystal violet staining of 24 h and 7-day biofilms grown on tissue culture plates in either 7H9 bacterial culture medium or cBD epithelial cell culture medium. N = 3 independent experiments. Statistics performed as two-way ANOVA with Tukey's correction for multiple testing. P<0.05 indicated with *, p<0.01 **, p<0.001 ***, p<0.0001 ****.

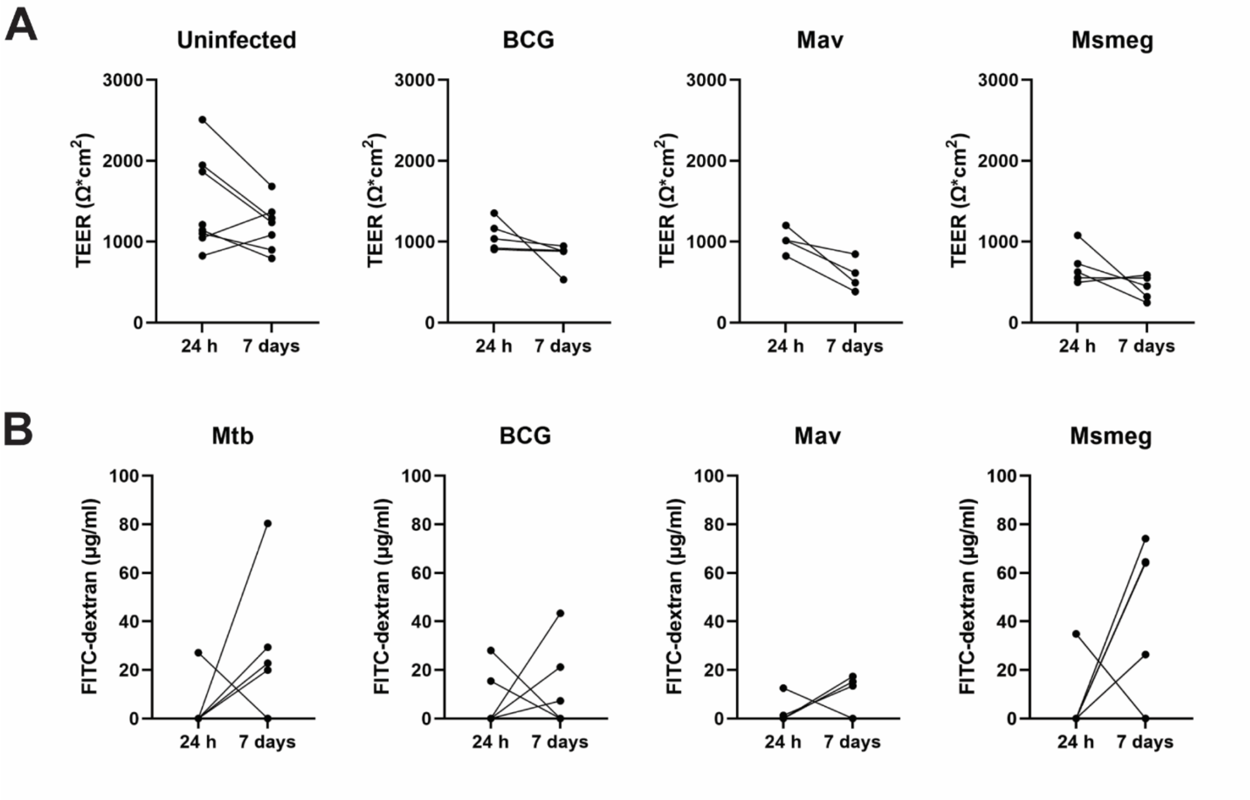

Figure S2. Magnitude of barrier disruption is partially dependent on host cells

Related to figure 4. (A) TEER measurements of PBEC after 24 h and 7 days biofilm formation, depicted per donor mix. N = at least 4 independent experiments using 4 different donor mixes. (B) FITC-dextran measurements after 24h and 7 days biofilm formation, depicted per donor mix. N = at least 4 independent experiments using 4 different donor mixes.

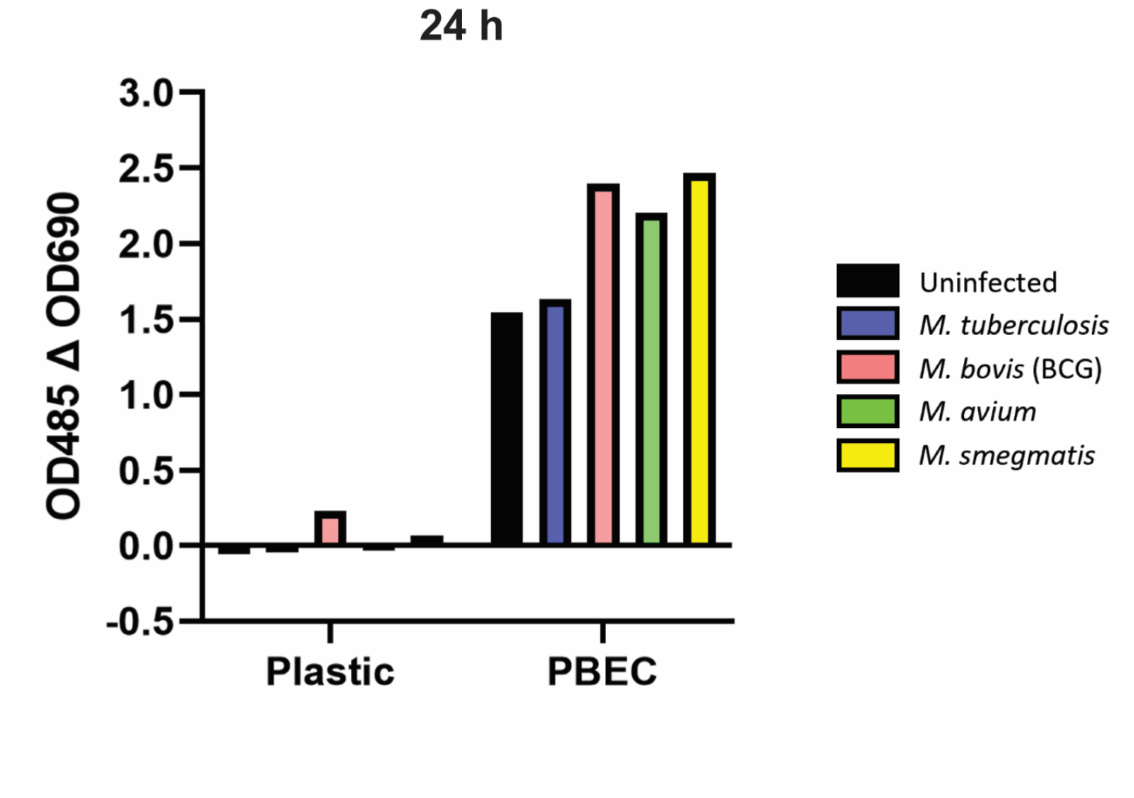

Figure S3. LDH release by mycobacteria alone is negligible

Related to figure 5. Optical density of LDH released by mycobacterial biofilms grown on plastic, and by PBEC infected with mycobacteria (as assessed in apical washes). Data from N = 1 experiment.

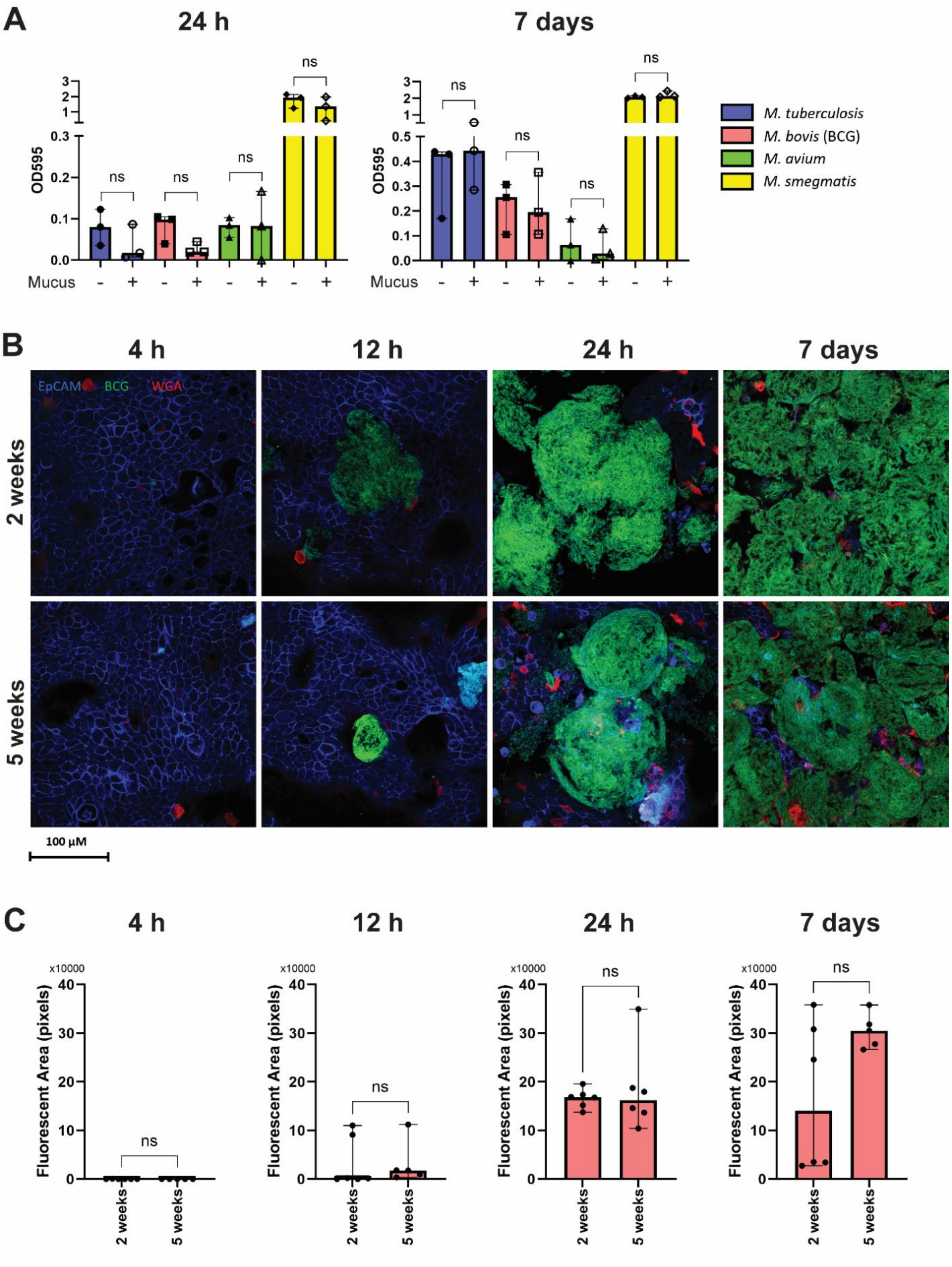

(Legend on next page)

Figure S4. Effects of mucus and longer PBEC differentiation on biofilm formation

Related to figure 6. (A) Bar graphs showing optical density of crystal violet staining of 24 h and 7-day biofilms grown in the presence or absence of mucus from uninfected donor pools. Data shown as median with range. Statistics performed as two-way ANOVA with Tukey's correction for multiple testing. P<0.05 indicated with *, p<0.01 **, p<0.001 ***, p<0.0001 ****.

(B) Confocal microscopy images of formation of biofilms and the secretion of polysaccharides in the biofilm matrix at 4 h, 12 h, 24 h and 7 days, on PBEC that were air exposed 2 weeks and 5 weeks prior to infection with BCG. Images from N = 2 independent experiments. EpCAM depicted in blue, bacteria in green, WGA in red. (C) Bar graphs depicting the total area in pixels which was positive for fluorescent bacteria. Data shown as median with range. Data points are single-plane images of z-stacks. Per experiment, the 3 planes with largest area of bacteria were chosen for quantification. Statistics performed as Friedman test with Dunn's correction for multiple testing. P<0.05 indicated with *, p<0.01 **, p<0.001 ***, p<0.0001 ****.

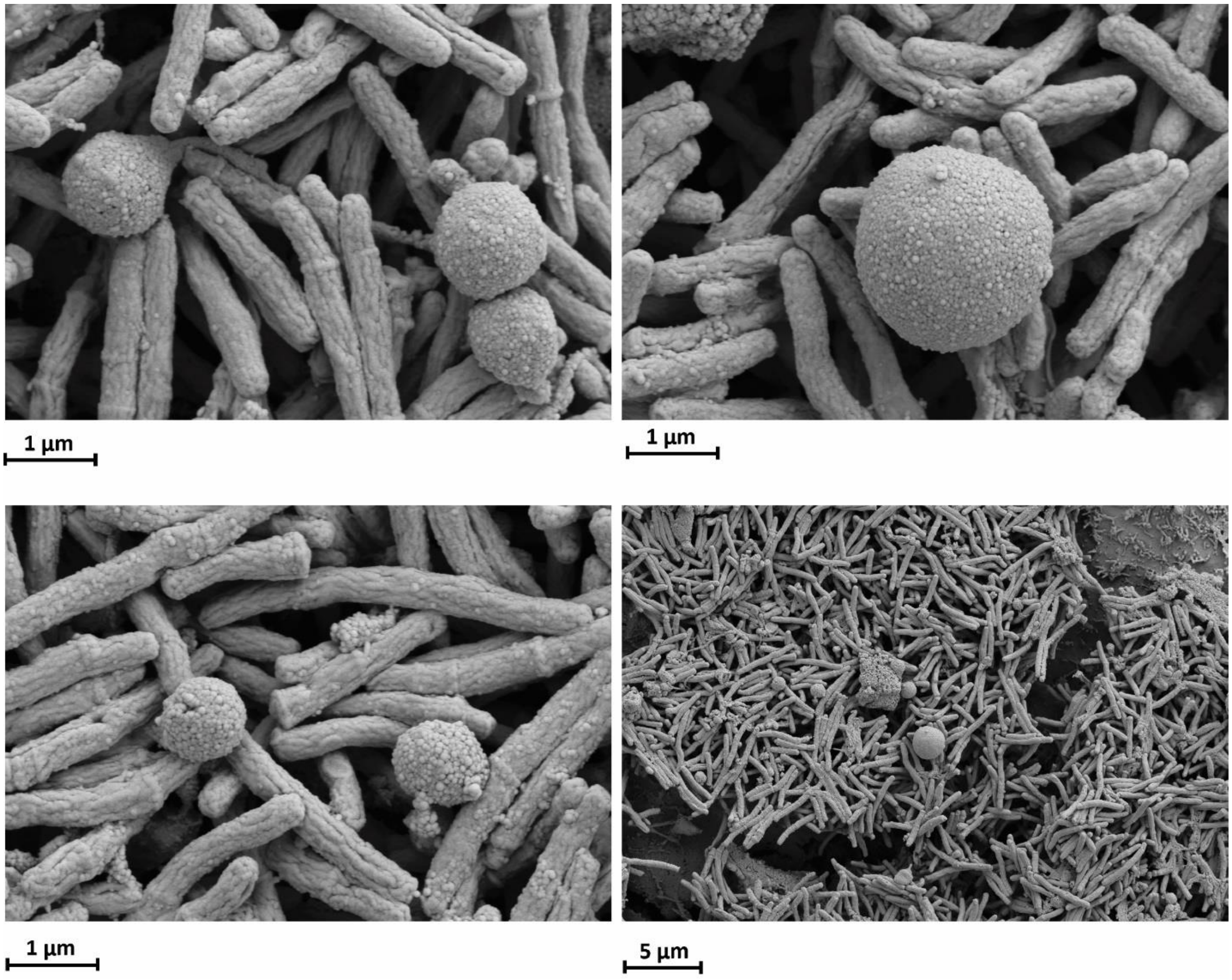

Figure S5. Ball-shaped forms observed in Mtb biofilms
Related to figure 7. Scanning electron microscopy images of ball-shaped forms in Mtb biofilms grown on PBEC for 24 hours. Images taken at 25,000 – 150,000 x magnification.

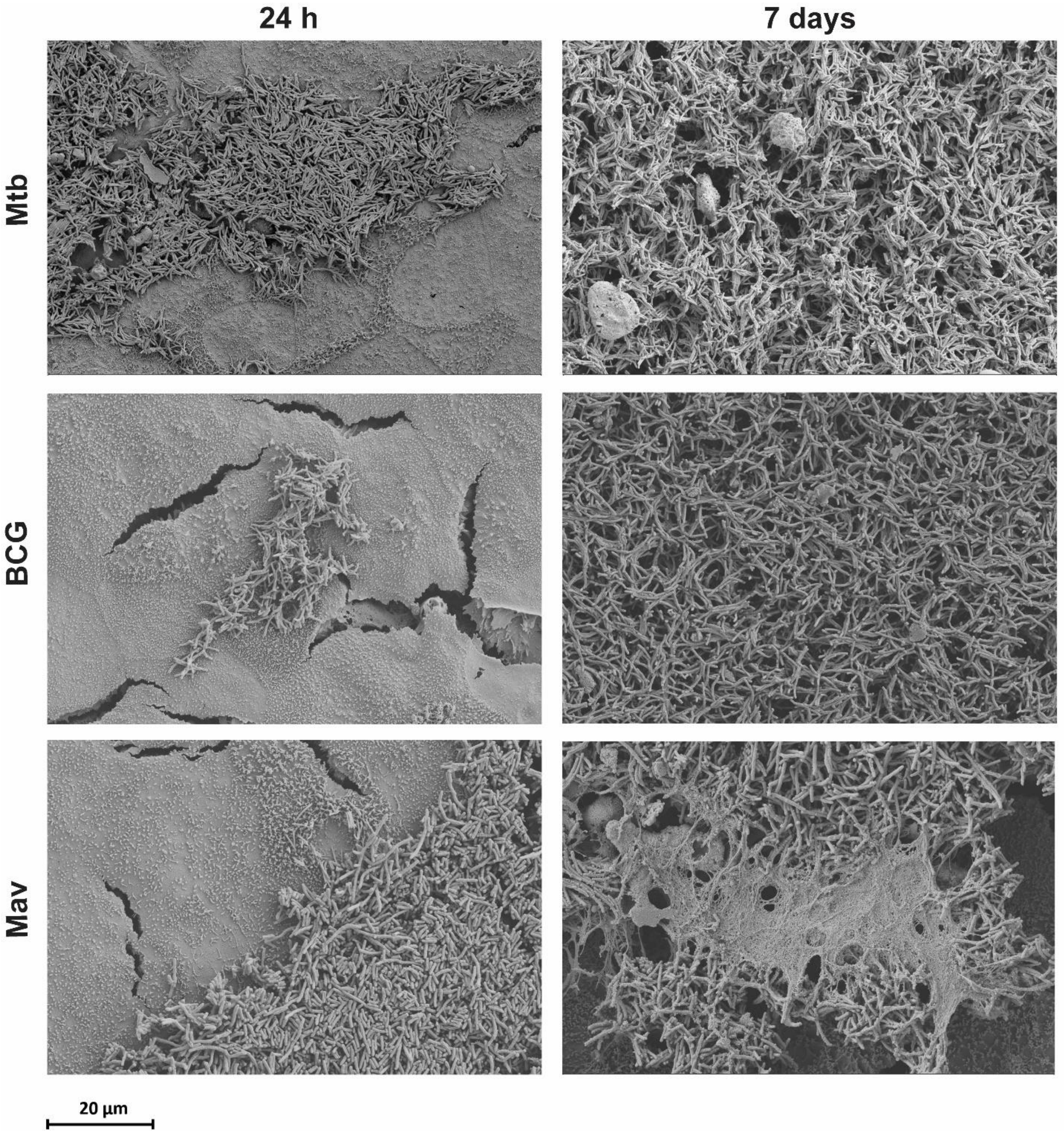

Figure S6. Increased structural organization of mycobacterial biofilms upon maturation
Related to figure 7. Scanning electron microscopy images of biofilms grown on PBEC for 24 h and 7 days showing a higher degree of organization at 7 days, as indicated by the formation of pore-like structures. Images taken at 10,000 x magnification.
